# Design and Characterisation of a Polyvinyl Chloride (PVC) Tissue-Mimicking Polymer Phantom for Quantitative Shear Wave Elastography Validation

**DOI:** 10.3390/polym18070797

**Published:** 2026-03-26

**Authors:** Wadhhah Aldehani, Sarah Louise Savaridas, Cheng Wei, Luigi Manfredi

**Affiliations:** 1 Division of Population Health and Genomics, School of Medicine, University of Dundee, Dundee DD1 9SY, UK; 2478077@dundee.ac.uk (W.A.); s.savaridas@dundee.ac.uk (S.L.S.); 2 Department of Clinical Radiology, Al-Sabah Hospital, Ministry of Health, Kuwait City 6347, Kuwait; 3 Breast Screening and Imaging Department, Ninewells Hospital, Dundee DD1 9SY, UK; 4 Biomedical Engineering, School of Science and Engineering, Fulton Building, University of Dundee, Dundee DD1 4HN, UK; c.wei@dundee.ac.uk; 5 Division of Respiratory Medicine and Gastroenterology, School of Medicine, University of Dundee, Dundee DD1 4HN, UK

**Keywords:** polyvinyl chloride (PVC), biomedical polymers, tissue-mimicking materials, breast phantom, shear wave elastography, ultrasound elastography, inter-operator variability, biomaterials for medical imaging

## Abstract

A polyvinyl chloride (PVC)-based tissue-mimicking polymer phantom was developed and mechanically characterised to replicate stiffness ranges relevant to breast elastography and to provide a controlled platform for evaluating shear wave elastography (SWE) measurements. SWE provides quantitative stiffness information that complements B-mode ultrasound in breast imaging. However, measurement variability related to operator technique and tissue continues to limit confidence in clinical interpretation. This study evaluates the reproducibility of SWE using custom-fabricated PVC-based breast phantoms with mechanically defined stiffness properties. Two PVC-based breast phantoms with identical geometry and different background stiffnesses were scanned using a single ultrasound system under a fixed SWE protocol. Each phantom contained four embedded inclusions representing clinically relevant stiffness categories. Six breast imagers independently acquired repeated SWE measurements in transverse and longitudinal planes, blinded to lesion identity and ground truth. Inter-operator reproducibility was assessed using intraclass correlation coefficients, and was high across both phantom backgrounds, with low intra-operator variability following quality assurance exclusion of one dataset due to sampling error. Measurement variability was lowest for solid inclusions and increased for the cyst-like inclusion in the stiffer background. SWE measurements consistently preserved the relative stiffness ordering of inclusions, although absolute values differed systematically from mechanically derived ground-truth stiffness. These findings demonstrate that PVC-based polymer phantoms provide a stable and reproducible platform for evaluating SWE measurement behaviour under controlled conditions. By isolating operator and acquisition effects from biological variability, this polymer-based framework supports methodological standardisation and structured operator training in breast elastography.

## 1. Introduction

Ultrasound elastography is becoming an increasingly important tool when it comes to diagnosing breast cancer. It complements traditional B-mode ultrasound, adding useful information regarding tissue stiffness [1]. Malignant breast lesions tend to demonstrate higher shear wave stiffness than benign masses. Clinical studies indicate that shear wave elastography (SWE) may improve the sensitivity, specificity and overall diagnostic accuracy of ultrasound for breast lesions, and may reduce benign biopsy rates [1,2,3,4,5,6]. Meta-analyses of SWE report pooled sensitivities around 85–94% and specificities in the range of 80–85% (depending on elasticity metric and cut-off), comparable to or better than conventional ultrasound alone [4,7].

However, shear wave elastography is not universally used in clinical practice despite increasing availability. The variability of elasticity measurements, which can arise from multiple technical and operator-related factors, remains a clinical concern. Probe pressure, transducer orientation, region of interest (ROI) placement, lesion depth, and local tissue stiffness all influence shear wave propagation. In addition, ultrasound manufacturers use proprietary reconstruction algorithms, resulting in cross-platform variability even when scanning the same lesion. This measurement inconsistency complicates the use of fixed diagnostic cut-offs and limits the broader standardisation of SWE in clinical practice [5,8,9].

Phantom studies provide a controlled environment for standardised testing, minimising the biological and anatomical variability that complicates in vivo breast imaging. Recent methodological work on tissue-mimicking materials and breast-specific phantoms has highlighted that relatively few published models combine rigorous mechanical characterisation, temporal stability testing, and stiffness ranges that truly reflect benign and malignant breast lesions [10]. Although most previous breast SWE phantom investigations report good intra- and inter-observer agreement, most rely on commercial or simplified phantoms that offer limited stiffness contrast and do not capture the heterogeneity of real breast pathology. In parallel, early clinical breast SWE studies proposed numerical stiffness cut-offs in the order of 50–80 kPa to help distinguish benign from malignant masses, often with high sensitivity for fibroadenoma classification; however, subsequent work has demonstrated substantial variation in “optimal” thresholds across patient cohorts, scanners, and acquisition protocols. As a result, no single stiffness value has been consistently adopted in clinical practice [11,12,13,14,15]. Together, these observations emphasise both the importance of breast-specific phantoms for isolating sources of variability in SWE measurements, and the need for system- and context-specific calibration when interpreting quantitative breast SWE thresholds [10,12,16].

In this context, the present work focuses on the design and functional validation of polyvinyl chloride (PVC)-based tissue-mimicking polymer phantoms with mechanically defined stiffness properties. Shear wave elastography is employed here as a characterisation tool to interrogate the dynamic response of these polymer constructs under controlled conditions. The study therefore examines how polymer formulation, background matrix stiffness, and inclusion–matrix interactions influence measured elastographic behaviour and reproducibility.

To address these gaps, this study used custom-fabricated breast phantoms containing inclusions with SWE stiffness characteristics that mimic clinically relevant breast lesions: cyst, fibroadenoma, and malignant-mimicking targets, embedded within two phantoms with physiologically representative background stiffnesses. The primary aim was to quantify the inter-operator reproducibility of SWE measurements under controlled conditions. Secondary aims were to evaluate intra-operator repeatability, characterise agreement between SWE values and mechanical ground-truth stiffness, and examine the behaviour of both clinical and data-driven diagnostic thresholds within the phantom model. By isolating operator and system effects from biological variability, the study provides new insight into SWE measurement consistency, stiffness-dependent bias, and threshold behaviour, supporting the use of phantoms as a reproducible framework for calibration, training, and methodological evaluation in breast elastography.

## 2. Materials and Methods

### 2.1. Study Design and Setting

This prospective, controlled phantom validation study was conducted at the breast imaging unit, NHS Tayside, University of Dundee, following STARD 2015 reporting guidelines for diagnostic accuracy studies. The study was designed as a blinded, multi-operator assessment of SWE reproducibility and diagnostic threshold performance using custom-fabricated tissue-mimicking phantoms. Primary objectives include: (1) Quantifying inter-operator SWE measurement reproducibility using intraclass correlation coefficients; and (2) evaluating diagnostic accuracy of proposed (50 kPa) versus optimised data-driven thresholds. Ethical approval was not required in line with the NHS Health Research Authority (HRA) decision tool (13 November 2025; IRAS Project ID 366162) as no patients or identifiable clinical data were involved, and scanning was performed exclusively on phantoms. Staff participation was covered under the University of Dundee’s low-risk staff-volunteer ethics process.

### 2.2. Participants

Six breast imagers, both consultant radiologists and consultant radiographers, participated in the study. Their experience in breast ultrasound, including SWE, ranged from less than two years to over ten years [17,18,19].

To minimise bias, operators did not receive additional training, demonstrations, or feedback during the study. They were blinded to the phantom design, the location of lesions, and the ground truth stiffness values of the inclusions.

### 2.3. Polymer Formulation and Phantom Design

**Figure 1:**
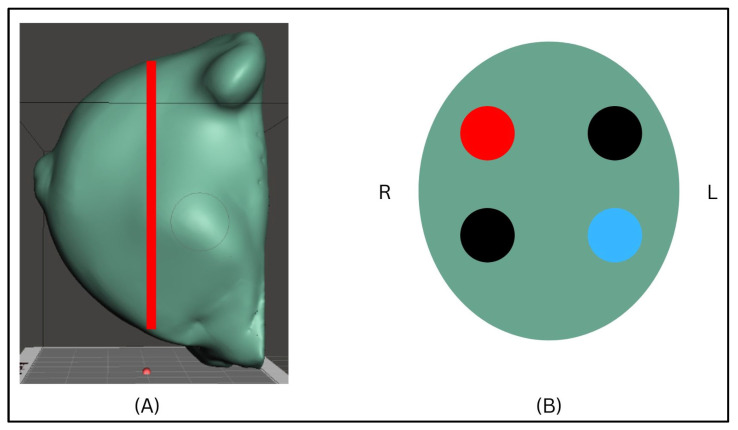
Design and experimental setup of the PVC-based breast phantom used in the study. (**A**) Three-dimensional model of the anatomically shaped breast phantom, illustrating the representative shear wave elastography (SWE) acquisition plane used during scanning. (**B**) Schematic top-view of the inclusion layout within the phantom, showing the relative positions and sizes of the four embedded inclusions; colour coding indicates stiffness category (blue = cyst-like, black = fibroadenoma-like and red = malignant-like).

Two breast-shaped phantoms with identical external geometry were fabricated using a PVC-based polymer composite designed as a tissue-mimicking material for ultrasound elastography applications. Each phantom contained four embedded inclusions positioned at a depth of 1 cm: one cyst-like inclusion, two fibroadenoma-like inclusions, and one malignant-mimicking inclusion (Figure 1). The spatial layout and inclusion order were identical in both phantoms to enable direct comparison of measurement behaviour across different background stiffness conditions.

PVC was selected as the base polymer due to its widespread use in ultrasound tissue-mimicking materials and its favourable mechanical stability when combined with plasticizers. Previous phantom development studies have demonstrated that PVC-based composites can reproduce the acoustic and mechanical properties of soft tissues used in ultrasound imaging applications [10]. The addition of a plasticizer reduces intermolecular interactions within the PVC matrix, allowing the elastic modulus of the composite to be tuned across a wide stiffness range relevant to breast tissue. Graphite powder was incorporated as an acoustic scatterer to generate ultrasound speckle patterns and adjust acoustic attenuation. The formulation and mechanical characterisation of this PVC-based composite system have been previously reported [20].

One phantom used a soft background density to mimic a predominantly fatty breast: the other a stiff background to mimic dense fibroglandular breast tissue. Components were hot-mixed with continuous stirring, degassed, cast into pre-treated moulds, cooled to room temperature, and demoulded. Full formulations and process parameters are listed in Table 1.

The stiffness of each phantom inclusion was controlled by adjusting the PVC–plasticizer ratio within the polymer structure. Increasing the PVC fraction increases polymer chain density and intermolecular interactions, resulting in a higher elastic modulus, whereas increasing the plasticizer fraction produces a softer material by increasing chain mobility. Using this principle, formulations were selected to reproduce the stiffness range associated with clinically relevant breast tissues, from cyst-like soft regions to high-stiffness malignant-mimicking inclusions. The selected formulations therefore allowed the fabrication of inclusions spanning the elastographic range typically encountered in breast SWE examinations. Mechanical compression testing and acoustic measurements of the PVC composite formulations confirmed that the resulting materials spanned the stiffness and acoustic ranges reported for breast tissues in the literature, validating their suitability for use as elastography phantoms [20].

**Figure 2:**
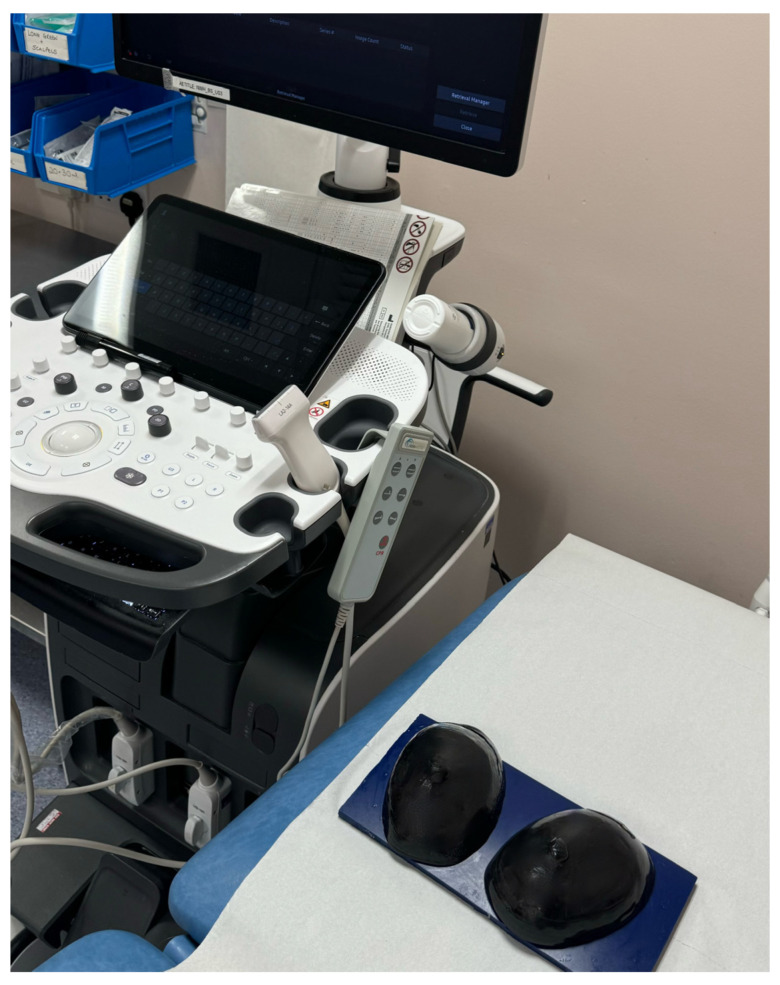
Experimental setup for SWE acquisition, showing the fabricated PVC-based breast phantoms positioned on the scanning platform and the ultrasound system used for operator-driven measurements under a standardised acquisition protocol.

The final fabricated PVC-based breast phantoms and the experimental scanning setup are provided in Figure 2. Following fabrication, the phantoms were stored at room temperature (20–22 °C) in sealed, opaque containers to minimise exposure to light, dust, and dehydration, and were maintained in a stress-free condition to avoid mechanical deformation. The phantoms were visually inspected before each scanning session, with no evidence of surface degradation, cracking, or physical change observed over the study period. SWE measurements were conducted across multiple scanning sessions using the same phantoms and standardised acquisition protocol, such that inter- and intra-operator repeatability metrics reflected both repeated measurements and short-term temporal stability of the polymer materials. While the present study was not designed as a long-term ageing or shelf-life evaluation, no systematic drift in SWE measurements was observed over the duration of the experimental trial.

### 2.4. Phantom Mechanical Property Testing

Mechanical and acoustic characterisation of the PVC–plasticizer–graphite composite formulations was performed to determine whether the fabricated materials reproduced the stiffness and acoustic ranges of breast tissues. Mechanical properties were assessed using uniaxial compression testing on an Instron 5567 universal testing machine equipped with a 1 kN load cell. Cubic specimens (40 × 40 × 40 mm) were compressed at a crosshead speed of 1 mm min^−1^ under quasi-static conditions. Young’s modulus was calculated from the linear region of the stress–strain curve (0–5% strain). For each formulation, three replicate tests were performed and averaged (Table 2).

### 2.5. Ultrasound System and Scanning Protocol

All acquisitions were performed using a Samsung HS50 ultrasound system (Seoul, Republic of Korea) equipped with a linear array (5–12 MHz) and quantitative SWE capability. Each operator scanned eight targets (four lesions in each of two phantoms) in both transverse and longitudinal planes. For each inclusion, two SWE measurements were acquired in the transverse plane and two in the longitudinal plane, resulting in four acquisitions per inclusion. This yielded 32 acquisitions per operator (eight inclusions × four acquisitions) and 192 total across all operators.

A circular Q-box ROI (2 mm) was placed over the visually stiffest portion of each inclusion. Following standard breast SWE practice, the mean SWE (kPa) within the ROI was recorded. B-mode frames were saved for quality control. Figure 2 shows the experimental setup.

In shear wave elastography, tissue stiffness is estimated from the measured shear wave propagation speed under the assumption of a linear, homogeneous, isotropic, and predominantly elastic medium. The reported stiffness values are derived from the shear modulus, calculated as $\mu =pc^{2}$, where p is the assumed material density and c is the measured shear wave speed, and are commonly expressed as an equivalent Young’s modulus using E≈3μ [21].The clinical SWE implementation used in this study does not explicitly model viscoelastic effects; viscosity-related dispersion and damping are therefore implicitly incorporated into the measured shear wave speed rather than independently quantified.

### 2.6. Quality Assurance of Operator Performance

After data collection, all SWE acquisitions were retrospectively reviewed for protocol adherence. For each operator and lesion, we examined B-mode, SWE settling time, and ROI placement.

Across operators, SWE measurements were generally consistent. However, for one operator (OP02), a marked variation was identified for a single inclusion. While OP02’s measurements were comparable to those of other operators for the majority of inclusions, two lesions demonstrated dramatic outlier values. Notably, the SWE values recorded by OP02 for those inclusions were closely aligned with those consistently obtained by all other operators for another lesion, rather than the intended target. This disparity raised concerns that an incorrect inclusion may have been sampled during acquisition.

Two design characteristics of the phantom may potentially have contributed to this error. First, the greyscale appearance of the inclusions was intentionally homogeneous and therefore more similar than typically seen in clinical breast imaging, which increases the likelihood of lesion misidentification. Second, the background material of the phantom was substantially firmer than normal breast tissue, making it easier to detect the same inclusion from different surface locations and potentially misattribute the target lesion.

To assess the impact of this behaviour, inter-operator ICCs were initially computed, including OP02. In Phantom 1, the ICC (C,1) was 0.95 (95% CI 0.81–0.996), whereas in Phantom 2, the ICC decreased to 0.82 (95% CI 0.48–0.98), with noticeable widening of the confidence intervals. Operator-level mean SWE values further demonstrated systematic differences for OP02, driven primarily by the outlying inclusions. Due to the lesion-specific nature of this variation, OP02 was excluded from the primary inter-operator reproducibility analysis. All primary results therefore reflect agreement across the five operators whose acquisitions fully adhered to the study protocol.

## 3. Results

Following the quality assurance review described earlier, one operator’s dataset was excluded from the primary analysis. This exclusion was driven by a lesion-specific outlier consistent with incorrect target sampling, which reduced inter-operator agreement when included. All primary analyses therefore report results from the five operators demonstrating consistent lesion localization.

All analyses were performed in R (version 4.3.0). For each operator and lesion, the four repeated SWE measurements were averaged to generate a single mean value for inter-operator analyses. Intra-operator analyses used all four individual readings.

### 3.1. Phantom Validation

Phantom validation demonstrated that the four fabricated inclusions (cyst, fibroadenoma 1 (FA1), fibroadenoma 2 (FA2), and malignant) produced distinct and well-separated stiffness distributions in both phantom backgrounds. In the P1 (fatty-like) phantom, the mean measured SWE values increased linearly with mechanical ground truth: cyst-like (18 ± 1.62 kPa; ground truth 26.1 kPa), FA1 (84.8 ± 8.31 kPa; 44.0 kPa), FA2 (154.0 ± 4.81 kPa; 52.8 kPa), and malignant (212.0 ± 10.9 kPa; 246.2 kPa).

A similar linear pattern was observed in P2 (glandular-like): cyst-like (27.6 ± 12.3 kPa), FA1 (69.5 ± 6.85 kPa), FA2 (127.0 ± 21.6 kPa), and malignant (203.0 ± 13.8 kPa), as reported in Table 2 and Table 3. Although FA2 had a benign ground-truth stiffness of 52.8 kPa, SWE values were consistently higher than expected in both backgrounds. FA2 showed the greatest inflation because its stiffness sat in the mid-range of the system’s sensitive region, where SWE is most susceptible to artefacts caused by background–lesion contrasts and shear wave interference. Unlike the cyst (very soft) and the malignant inclusion (very stiff), FA2 lies close enough to the background stiffness that even minor probe pressure, slight ROI mis-centring, or partial sampling of the lesion boundary significantly elevates the reconstructed mean shear wave elastography. In addition, FA2 is large enough to produce internal shear wave reflection patterns but not stiff enough to damp them, which further causes overestimation. These effects are far less prominent in the cyst (minimal shear wave propagation) and in the malignant inclusion (high stiffness dominates the reconstruction), making FA2 uniquely sensitive to upward bias.

Furthermore, no overlap occurred between the benign inclusions (cyst/FA1/FA2) and the malignant inclusion in either phantom, confirming that the designed mechanical stiffness hierarchy was accurately reproduced in the SWE measurements. One-way ANOVA demonstrated highly significant differences among the four lesion types for both phantoms (P1: *F*(3, 16) = 656.7, *p* < 0.001; P2: *F*(3, 16) = 135.4, *p* < 0.001), supporting the robustness and discriminative validity of the phantom materials.

### 3.2. Inter-Operator Reliability

**Figure 3:**
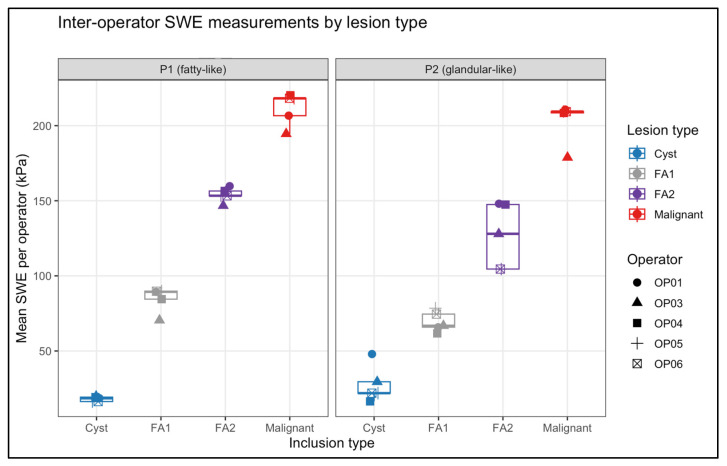
Operator-level mean SWE values per lesion for both phantoms. Each point represents the average of four SWE acquisitions for one operator. Boxplots summarise inter-operator variability. High clustering across operators reflects excellent reproducibility (ICC P1 = 0.992; P2 = 0.964).

Inter-operator reliability was evaluated using lesion-level mean SWE values for each operator (Table 4). As illustrated in Figure 3, operator-specific means clustered tightly within each lesion type in both phantom backgrounds, demonstrating minimal dispersion relative to the wide stiffness range represented by the inclusions.

When OP02 was included in the analysis, inter-operator reliability remained high in the fatty-like phantom (ICC(C, 1) = 0.95; 95% CI 0.81–0.996) but fell to 0.82 (95% CI 0.48–0.98) in the glandular-like phantom. This corresponded to substantially larger between-operator spread in P2, with OP02’s lesion-specific means deviating by up to 60–80 kPa from the other operators for the FA1 and FA2 inclusions. In addition, OP02 exhibited the largest within-operator dispersion in P2 (SD ≈ 10–11 kPa for the FA1 and malignant inclusions). These findings support the decision to exclude OP02 from the primary analysis and to base reproducibility estimates on the five operators whose measurements were stable and protocol compliant.

Operator variability was quantified using the coefficient of variation (CV%), summarised per lesion type in Table 5. In the P1 (fatty-like) phantom, inter-operator CVs were low across all inclusions, ranging from 3.1% for FA2 to 9.8% for FA1, with the cyst-like and malignant inclusions showing similarly small variability (9.0% and 5.2%, respectively). In the P2 (glandular-like) phantom, the three solid inclusions again demonstrated low variability, with CVs of 9.9% (FA1), 17.1% (FA2), and 6.8% (malignant). In contrast, the P2 cyst-like inclusion showed markedly higher variability (CV 44.7%), consistent with the known susceptibility of very soft structures embedded in stiff backgrounds to small acquisition differences.

Phantom-level inter-operator reliability was excellent for both backgrounds (Table 6). ICC (2, 1) was 0.995 (95% CI 0.978–1.000) for P1 and 0.963 (95% CI 0.851–0.997) for P2, indicating that 96–99% of measurement variance reflected true lesion stiffness rather than operator differences. Together, the low lesion-specific CVs, high ICC values, and visually compact operator distributions in Figure 3 confirm that SWE measurements were highly reproducible across operators, with only the very soft P2 cyst showing notable operator-dependent variability.

### 3.3. Intra-Operator Repeatability

Intra-operator repeatability was assessed using the SD of the four repeated SWE acquisitions for each lesion and operator (Table 7). Across both phantoms, variability remained low, relative to the overall stiffness range of the inclusions, with generally smaller dispersion in the stiffer background. In P1, the median intra-operator CV across all lesion types and operators was 14.1% (IQR 10.6–17.4%), with lesion-specific medians of 13.4% for cyst-like, 16.6% for FA1, 13.3% for FA2, and 14.1% for malignant inclusions. In P2, the median intra-operator CV was 8.3% (IQR 5.7–16.8%), again with lower variability for higher-stiffness targets; lesion-specific medians were 16.3% for cyst-like, 18.0% for FA1, 6.9% for FA2, and 6.6% for malignant inclusions. Across both backgrounds, CVs were highest for FA1 and lowest for malignant lesions, consistent with broader SWE distribution in softer tissue-mimicking inclusions and tighter clustering at higher stiffness values. Overall, the distribution of CVs indicates good within-operator precision, with the majority of lesion-level repeatability estimates falling below 20% in both phantoms.

### 3.4. Agreement Between SWE Measurements and Mechanical Ground Truth

**Figure 4:**
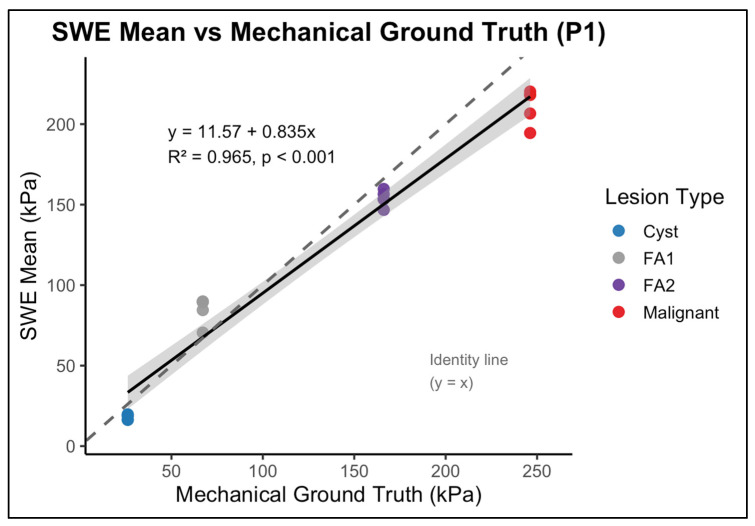
Linear regression between shear wave elastography (SWE)-derived mean stiffness and mechanically measured ground-truth stiffness for inclusions embedded in the P1 (fatty-like background) phantom. Each point represents an operator-specific mean SWE measurement for an individual inclusion. The solid line indicates the fitted regression with 95% confidence interval (shaded), while the dashed line represents the line of identity (y = x). SWE measurements demonstrate a strong linear relationship with mechanical stiffness while exhibiting systematic deviation from absolute equivalence.

**Figure 5:**
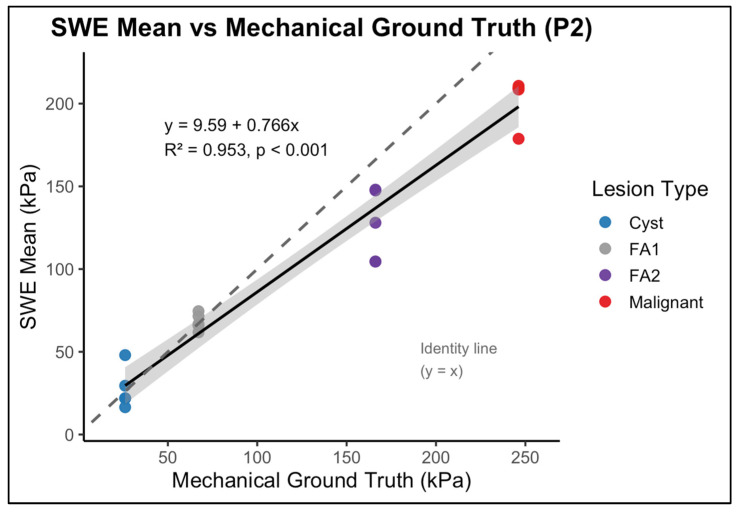
Linear regression between shear wave elastography (SWE)-derived mean stiffness and mechanically measured ground-truth stiffness for inclusions embedded in the P2 (glandular-like background) phantom. Each point represents an operator-specific mean SWE measurement for an individual inclusion. The solid line indicates the fitted regression with 95% confidence interval (shaded), and the dashed line represents the line of identity (y = x). Compared with P1, the stiffer background results in greater compression of SWE-derived values at higher stiffness levels, reflecting increased background-dependent measurement bias.

The relationship between SWE measurements and mechanical ground-truth stiffness was evaluated for each phantom background using operator-level mean SWE values for the four inclusion types (Figure 4 and Figure 5). As expected, the SWE values did not numerically match the mechanical reference. Instead, a non-linear bias pattern was observed across the stiffness spectrum. Intermediate-stiffness inclusions (FA1: 44 kPa; FA2: 52.8 kPa) were substantially overestimated in both phantoms, whereas the highest stiffness inclusion (malignant, 246.2 kPa) was consistently underestimated. Cyst-like inclusion (26.1 kPa) was underestimated in P1 and showed variable agreement in P2.

Despite these deviations in absolute magnitude, stiffness ordering was preserved across inclusion types. Regression analysis demonstrated a strong liner association between operator-level mean SWE values and mechanical ground truth stiffness values in both phantoms. In P1, the relationship yielded $R^{2}=0.965\;(p\;<0.001)$ (Figure 4), and in P2, a value of $R^{2}=0.953\;\left(\;p\;<0.001\right)$ (Figure 5). These finding indicate that, although absolute values were systematically biassed and the dynamic range was compressed, relative stiffness ranking across inclusion remained stable. Thus, SWE preserved discrimination between low-, intermediate-, and high-stiffness targets despite magnitude distortion.

## 4. Discussion

This study evaluated shear wave elastography (SWE) performance in breast imaging using two custom-fabricated PVC-based phantoms containing four clinically relevant stiffness categories. By eliminating biological variability and applying a strictly standardised acquisition protocol, the study isolated the contributions of operator behaviour, background stiffness, and system response to SWE performance. The findings demonstrate exceptionally high reproducibility across operators and provide important insights into SWE stiffness accuracy and artefact behaviour within a controlled experimental setting.

A key outcome was the extremely high inter-operator reproducibility observed across both phantoms, with ICC values exceeding 0.96 and reaching 0.99 in the fatty-like background. These values outperform those reported in clinical SWE studies, where biological heterogeneity and inconsistent probe handling typically reduce reproducibility. For example, Cosgrove et al. and Evans et al. reported intra-operator ICCs of 0.84–0.87, while more variable performance has been identified in strain elastography and mixed cohorts, with ICCs as low as 0.25 [11,17]. Previous phantom studies report ICCs in the range of 0.75–0.77. The overall higher reliability in the present study confirms that SWE, when performed under tightly controlled conditions and using physically stable materials, is capable of near-perfect reproducibility [8,10,22].

In clinical shear wave elastography, stiffness is calculated from the measured shear wave speed using an elastic inversion model, where the shear modulus is proportional to the square of the measured wave speed. Consequently, any systematic underestimation of shear wave speed will directly produce a lower SWE-derived modulus [23,24]. Importantly, neglecting viscosity in the inversion does not impose a fixed bias direction; rather, the reported modulus depends primarily on the accuracy of the reconstructed wave speed. In this phantom study, the cyst-like and malignant inclusions represent the stiffness boundaries, where shear wave speed estimation is most vulnerable to systematic bias. The mechanical reference values were obtained from quasi-static uniaxial compression testing, reflecting low-frequency, boundary-constrained deformation. By contrast, SWE measures transient shear wave propagation within finite inclusions embedded in a background medium. These represent fundamentally different mechanical conditions, and numerical equivalence between the two methods is not theoretically required. When inclusion dimensions approach the shear wavelength, boundary interactions can alter local wave behaviour and reduce the effective wave speed estimated within the region of interest. In addition, reduced signal-to-noise in very soft targets and tracking or regularisation constraints in very stiff targets may further attenuate reconstructed wave speed. Given the quadratic relationship between wave speed and modulus, such reductions in measured speed are sufficient to account for the lower SWE-derived values observed at the stiffness extremes, even in the absence of explicit viscosity modelling.

While reproducibility was excellent, agreement between SWE measurements and mechanical ground truth presented a more complex pattern. Across both phantom backgrounds, SWE correctly preserved the logical stiffness hierarchy, cyst-like < FA1 < FA2 < malignant, which indicates that the system’s ability to differentiate between lesion stiffness categories was maintained. However, absolute stiffness values deviated from the mechanical reference, which reflects a combination of system limitations and well-recognised SWE artefacts. This behaviour is illustrated in Figure 4 and Figure 5, which shows the relationship between SWE-derived stiffness and mechanical ground-truth values, including systematic deviation from the identity line despite preservation of relative stiffness ordering.

The malignant inclusion, designed at approximately 246 kPa, was consistently underestimated. This behaviour is reported in high-stiffness phantoms and reflects the upper performance limits of SWE systems. At high stiffness, shear waves accelerate and may exceed the tracking capabilities of the imaging platform, while increased attenuation and wavefront distortion further compress the reconstructed elasticity values [15,25].

In contrast, the softer inclusions, especially FA1 and FA2, with mechanical stiffnesses of 44 kPa and 52.8 kPa, respectively, were systematically overestimated. This inflation is likely multifactorial, arising from interactions between inclusion and background stiffness, partial-volume effect at lesion boundaries, and increased sensitivity to wave reflection and interference within the mid-range stiffness level. FA2 showed the largest difference, consistent with its location in the stiffness “sensitive region” where small changes in propagation conditions lead to substantial variation in measured elasticity [26,27,28,29].

**Figure 6:**
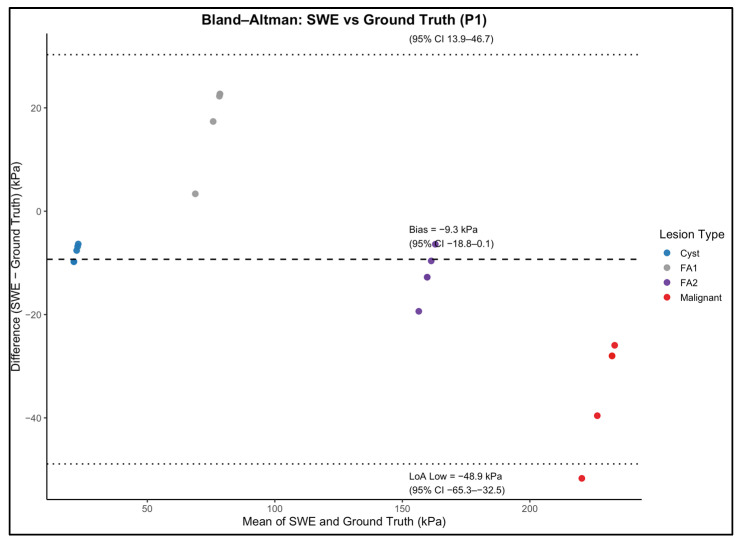
Bland–Altman analysis comparing shear wave elastography (SWE)-derived stiffness with mechanically measured ground-truth stiffness for inclusions embedded in the P1 (fatty-like background) phantom. Each point represents an operator-specific mean SWE measurement for an individual inclusion. The dashed line indicates the mean bias, and the dotted lines represent the 95% limits of agreement (LoA). The distribution demonstrates modest systematic underestimation of stiffness with relatively narrow agreement limits, consistent with the softer background material.

**Figure 7:**
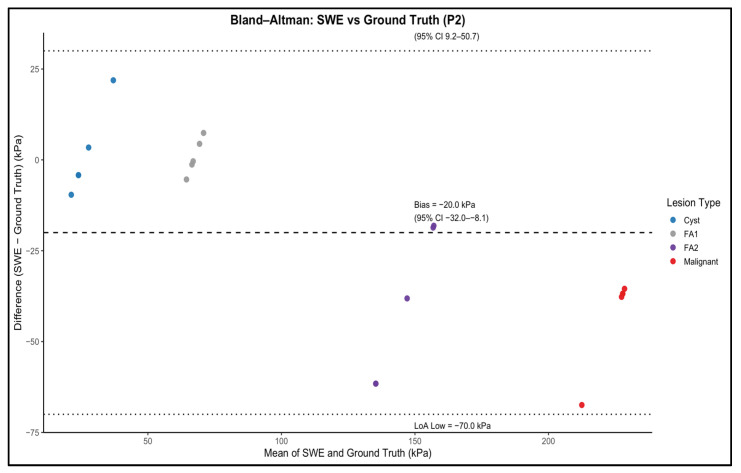
Bland–Altman analysis comparing shear wave elastography (SWE)-derived stiffness with mechanically measured ground-truth stiffness for inclusions embedded in the P2 (glandular-like background) phantom. Each point represents an operator-specific mean SWE measurement for an individual inclusion. The dashed line indicates the mean bias, and the dotted lines represent the 95% limits of agreement (LoA). Compared with P1, the stiffer background produces increased negative bias and wider agreement limits, particularly for higher-stiffness inclusions.

Beyond system-specific limitations, the observed difference between SWE-derived stiffness estimates and mechanically derived Young’s modulus values should be interpreted in the framework of the fundamentally different force scenarios and material responses investigated by these techniques. Mechanical compression testing provides a quasi-static measure of elastic behaviour under low-frequency deformation, whereas shear wave elastography interrogates the material response under dynamic, high-frequency shear wave propagation. For viscoelastic materials such as PVC–plasticiser composites, the effective stiffness is frequency-dependent and influenced by viscous damping and energy transfer mechanisms. As a result, direct numerical equivalence between static mechanical measurements and dynamic elastography-derived values should not be expected, even under idealised conditions. The systematic offsets observed in this study are therefore consistent with known viscoelastic behaviour rather than indicative of measurement error or material instability. Furthermore, while absolute values differed, SWE measurements preserved relative stiffness ordering between inclusions, supporting the suitability of the phantom for comparative, reproducibility, and operator-performance studies rather than absolute modulus calibration [30,31]. The magnitude and direction of this systematic bias across the stiffness range are further demonstrated by the Bland–Altman analysis shown in Figure 6 and Figure 7.

These findings have implications for future phantom design. Ground-truth stiffness values derived from isolated mechanical testing may not directly translate to the effective stiffness measured for an embedded inclusion under SWE. Incorporating adjustments for background–inclusion interactions, or calibrating phantom targets against in vivo reference behaviour rather than isolated material properties, may improve the physiological realism and interpretability of future elastography phantoms.

Such compensation could be achieved by characterising inclusions within representative background materials and defining effective stiffness ranges rather than single absolute values. This approach would better reflect clinical measurement conditions, where lesion stiffness is directly influenced by surrounding tissue properties, probe loading, and wave propagation conditions, and may support a more robust cross-platform calibration of SWE systems.

The interpretation of linearity between measured and mechanical stiffness must therefore be approached cautiously. Although the measured values increased linearly with ground truth, the wide separation between the benign and malignant mechanical targets limits the use of treating the relationship as a strict linear calibration curve. The findings instead reinforce a more clinically aligned perspective. SWE behaves reliably in terms of categorical classification, but absolute values are influenced by acquisition conditions, background mechanics, and the physical limitations of wave propagation. This aligns with clinical experience, where thresholds around 50 kPa differentiate benign and malignant lesions not because of exact material stiffness, but because of consistent physiological contrasts under in vivo conditions.

Background stiffness also had a notable influence on measurement behaviour. The glandular-like phantom produced higher operator-dependent variability than the fatty-like phantom, despite both phantoms being mechanically homogeneous. This pattern mirrors clinical observations in dense breast tissue, where increased stiffness, acoustic backscatter, and complex internal interfaces are associated with greater variability in SWE measurements. The present findings indicate that elevated baseline stiffness alone is sufficient to reduce SWE stability, highlighting the importance of the mechanical context in which lesions are embedded.

This observation has direct clinical relevance. It suggests that SWE-based thresholds used to differentiate benign from malignant lesions may be influenced by background breast density, an effect that is not routinely accounted for in current clinical practice [15]. This consideration is particularly important in younger women, who typically have denser breast tissue [32,33] and represent the primary population in which biopsy avoidance strategies are proposed [34,35,36]. Incorporating background tissue characteristics into SWE interpretation, or developing density-aware diagnostic thresholds, warrants further investigation to improve the reliability and clinical applicability of elastography-based decision-making.

Intra-operator repeatability further supported the stability of the SWE acquisition protocol. Median coefficients of variation were low in the fatty-like background and modestly higher in the glandular-like background, consistent with the expected influence of tissue mechanics on shear wave propagation. The combined reproducibility and repeatability results indicate that operators performed consistently and that remaining variability primarily reflects system-level and physical factors [12,37]. Operator training plays a crucial role in reducing variability in SWE measurements. By ensuring that operators are well-versed in standardised acquisition techniques and aware of the influence of background mechanics, training can help mitigate the effects of operator-dependent variability. This emphasises the need for comprehensive training programmes to enhance the reliability and accuracy of SWE in clinical settings [37,38,39,40].

Overall, this study demonstrates that high-fidelity PVC-based phantoms provide an effective platform for evaluating SWE reproducibility, characterising artefact behaviour, and supporting calibration. The findings show that SWE achieves excellent reproducibility under controlled conditions, but that absolute stiffness interpretation remains dependent on system physics, background stiffness, and lesion mechanics. These insights reinforce the value of phantom-based studies in complementing clinical data and supporting the responsible use of SWE in breast imaging.

## 5. Limitations

First, this is a phantom study; although phantoms allow known ground truth and strict control, they cannot reproduce the heterogeneity of human tissue. Second, only a single vendor/platform and acquisition preset were tested; thresholds and biases may differ across systems. Third, lesion depths here were shallow; depth-dependent attenuation and decorrelation may alter accuracy in vivo. Finally, the malignant set included a small number at the lower stiffness boundary, which influences optimal threshold placement and confidence intervals.

## 6. Future Work

Future work should expand the phantom design to more closely replicate the complexity of breast tissue. Incorporating heterogeneous inclusions, multi-layered backgrounds, and irregular lesion geometries would improve anatomical realism and allow for a more nuanced assessment of SWE performance in challenging scenarios. Cross-vendor comparison studies are also warranted, as variability in reconstruction algorithms, push-pulse parameters, and quality-mapping strategies may alter both stiffness accuracy and reproducibility. Additionally, integrating embedded sensors or pressure-monitoring systems could help quantify compression effects and further reduce operator-dependent variability. Investigating temporal stability and long-term durability of the PVC-based formulations would also support their use in routine quality assurance. Finally, pairing phantom evaluation with parallel clinical data, particularly for small, deep, or low-contrast lesions, may help translate phantom-derived insights into practical guidance for SWE interpretation and training.

## 7. Conclusions

This study demonstrates that, under controlled conditions with minimised biological and operator variability, shear wave elastography applied to PVC-based breast phantoms exhibits high operator reproducibility. Absolute stiffness values did not numerically match quasi-static mechanical ground-truth measurements, reflecting differences between dynamic wave-based estimation and static compression testing, as well as known constraints in shear wave speed construction at stiffness extremes. However, relative stiffness ordering of inclusions was consistently preserved across phantoms with different background properties.

These findings support the use of PVC-based phantoms as stable and reproducible test objects for SWE method validation, operator training, and comparative system evaluation. As a result, study findings should be interpreted as establishing a controlled reference framework rather than replicating clinical complexity. This reinforces the need for continued improvement in phantom design and acquisition standardisation to better understand SWE measurements in real-world breast imaging.

## Figures and Tables

**Table 1:** Phantom material formulations.

Component	PVC (% *w*/*w*)	Plasticizer (% *w*/*w*)	Scatterer Type	Scatterer (% *w*/*w*)	Notes
Soft dense background (P1)	7.5	90.5	graphite	2	Fatty breast tissue mimic
Stiff dense background (P2)	8	90	graphite	2	Dense fibroglandular parenchyma mimic
Cyst	5	91	graphite	4	Cyst-like stiffness
Fibroadenoma inclusion	10	89.5	graphite	0.5	FA-like benign mimic
Fibroadenoma inclusion	10	88	graphite	2	FA-like benign mimic
Malignant inclusion	12	86	graphite	2	Malignant-like stiffness/heterogeneity

**Table 2:** Relationship between phantom inclusion stiffness ranges and commonly used clinical SWE diagnostic categories.

Inclusion Type (Phantom)	Ground Truth Stiffness (kPa)	Typical Clinical Interpretation
Cyst-like inclusion	26.1 Pa	Benign/low stiffness
Fibroadenoma-like (FA1)	44 kPa *	Benign/borderline
Fibroadenoma-like (FA2)	52.8 kPa *	Benign/borderline
Malignant-like	246.2 kPa	Highly suspicious malignant

* Clinical interpretation based on commonly reported SWE stiffness ranges in breast imaging literature; thresholds may vary by vendor and clinical protocol.

**Table 3:** Summary statistics for SWE measurements by phantom ground truth inclusion type. Ground truth values represent the designed mechanical properties of phantom inclusions.

Phantom	Clinical Diagnosis	SWE Mean ± SD (kPa)	Median (kPa)	Range (kPa)
P1	Cyst-like	18.0 ± 1.62	18.5	16.3–19.8
P2	Cyst-like	27.6 ± 12.3	21.9	16.5–48.0
P1	FA1 (Benign)	84.8 ± 8.31	89.4	70.5–89.8
P2	FA1 (Benign)	69.5 ± 6.85	66.8	61.8–78.5
P1	FA2 (Benign)	154.0 ± 4.81	153	147.0–160.0
P2	FA2 (Benign)	127.0 ± 21.6	128	105.0–148.0
P1	Malignant	212.0 ± 10.9	218	194.0–220.0
P2	Malignant	203.0 ± 13.8	209	179.0–211.0

Note. Values represent operator-level SWE mean stiffness (kPa), averaged across four repeated measurements per inclusion. Ground truth stiffness values were determined from mechanical compression testing. FA1 and FA2 represent two benign solid inclusions with distinct designed stiffness values (44.0 kPa and 52.8 kPa, respectively).

**Table 4:** Mean SWE (kPa) per lesion per operator.

Phantom	Lesion	OP01	OP03	OP04	OP05	OP06
P1	Cyst	18.5	19.8	19.2	16.3	16.3
	FA1	89.4	70.5	84.5	89.8	89.8
	FA2	160	174	156	153	153
	Malignant	207	194	220	218	218
P2	Cyst	48	29.5	16.5	21.9	21.9
	FA1	65.8	66.8	61.8	78.5	74.6
	FA2	148	128	148	105	105
	Malignant	211	179	208	209	209

Note: Values represent the mean SWE from four repeated acquisitions per operator for each lesion.

**Table 5:** Inter-operator variability per lesion (mean SWE, SD, CV%).

Phantom	Lesion Type	Mean SWE (kPa)	SD (kPa)	CV (%)
P1	Cyst	18.0	1.62	8.98
	FA1	84.8	8.31	9.80
	FA2	154.0	4.81	3.12
	Malignant	212.0	10.9	5.17
P2	Cyst	27.6	12.3	44.70
	FA1	69.5	6.85	9.85
	FA2	127.0	21.6	17.10
	Malignant	203.0	13.8	6.77

Note: CV = SD/mean × 100. Higher variability for the P2 cyst reflects known instability of very soft inclusions in stiff backgrounds.

**Table 6:** Inter-operator reliability (ICC (2, 1)) per phantom using lesion-level mean SWE values.

Phantom	ICC (2, 1)	95% CI	*p*-Value
P1	0.995	0.978–1.000	1.29 × 10^−14^
P2	0.963	0.851–0.997	1.89 × 10^−9^

Note: ICC values ≥ 0.90 indicate excellent inter-operator reliability. ICC computed using lesion-level operator means.

**Table 7:** Intra-operator repeatability expressed as the coefficient of variation (CV%) for each lesion type in both phantoms. Values represent lesion-level median CVs with corresponding ranges, and overall phantom variability is summarised using median and IQR.

Phantom	Lesion Type	Median CV (%)	Range (%)
P1 (fatty-like)	Cyst-like	13.4	8.8–28.0
	FA1	16.6	4.2–50.8
	FA2	13.3	6.9–27.6
	Malignant	14.1	8.8–27.6
P1 Overall	—	14.1	10.6–17.4 (IQR)
P2 (glandular-like)	Cyst-like	16.3	4.4–39.9
	FA1	18.0	3.5–39.9
	FA2	6.9	4.2–36.8
	Malignant	6.6	4.2–16.3
P2 Overall	—	8.3	5.7–16.8 (IQR)

## Data Availability

The datasets generated and analysed during the current study are available from the corresponding author on reasonable request.
